# Multimodality treatment of malignant pleural mesothelioma

**DOI:** 10.12688/f1000research.15796.1

**Published:** 2018-10-22

**Authors:** Lawek Berzenji, Paul Van Schil

**Affiliations:** 1 Department of Thoracic and Vascular Surgery, Antwerp University Hospital, Wilrijkstraat 10, B-2650 Edegem (Antwerp), Belgium

**Keywords:** Mesotheliomal, Multimodality treatment, Trimodality treatment

## Abstract

Malignant pleural mesothelioma (MPM) is a rare disease of the pleura and is largely related to asbestos exposure. Despite recent advancements in technologies and a greater understanding of the disease, the prognosis of MPM remains poor; the median overall survival rate is about 6 to 9 months in untreated patients. The main therapeutic strategies for MPM are surgery, chemotherapy, and radiation therapy (RT). The two main surgical approaches for MPM are extrapleural pneumonectomy (EPP), in which the lung is removed en bloc, and pleurectomy/decortication, in which the lung stays *in situ*. Chemotherapy usually consists of a platinum-based chemotherapy, such as cisplatin, often combined with a folate antimetabolite, such as pemetrexed. More recently, immunotherapy has emerged as a possible therapeutic strategy for MPM. Evidence suggests that single-modality treatments are not an effective therapeutic approach for MPM. Therefore, researchers have started to explore different multimodality treatment approaches, in which often combinations of surgery, chemotherapy, immunotherapy, and RT are investigated. There is still no definitive answer to the question of which multimodality treatment combinations are most effective in improving the poor prognosis of MPM. Research into the effects of trimodality treatment approaches have found that radical approaches such as EPP and hemithoracic RT post-EPP are less effective than was previously assumed. In general, there are still a great number of unanswered questions and unknown factors regarding the ideal treatment approach for MPM. Hopefully, more research into multimodality therapy will provide insight into which combination of treatment modalities is most effective.

## Introduction

Malignant mesothelioma is a rare malignant tumor arising from serosal surfaces that can affect the pleura, peritoneum, tunica vaginalis, and pericardium. The most common type is malignant pleural mesothelioma (MPM), which accounts for about 65% of all malignant mesotheliomas ^1, 2^. The three major histological subtypes are the epithelioid, sarcomatoid, and biphasic mesotheliomas, and the epithelioid type is the most prevalent ^3, 4^. For more ambiguous cases of biphasic mesotheliomas, the concept of a “transitional” subtype has been proposed. This subtype applies mainly to cases in which there is ambiguity in quantifying sarcomatoid components in biphasic mesotheliomas ^5^. MPM has a peak incidence in the fifth and sixth decades of life and is more common in men than in women ^6^. It has been shown that the majority of cases of MPM are related to exposure to amphibole asbestos. A number of other possible etiologies such as simian virus-40, upregulation of mitogen-activated protein kinases, and DNA damage by iron-related reactive oxygen species have also been investigated ^7^.

Although MPM is a relatively rare malignancy, the incidence has slightly increased over the last decade because of a lag time in tumor development of 30 to 50 years post-exposure to asbestos. The reported incidence is highest in industrialized countries (up to 30 cases per million), especially in countries such as the UK, Australia, and Belgium, where asbestos was widely used in many industries in the past ^8^. Although many developed countries have issued bans on using asbestos in professional environments, exposure to asbestos is still possible because of a rise in do-it-yourself renovations of homes containing asbestos ^9^. Data on the incidence of MPM in developing and newly industrialized countries are lacking and often difficult to obtain; however, available data show relatively higher incidences in countries such as Brazil, Russia, and China, where regulations around asbestos exposure are less strict ^10, 11^. Furthermore, the estimated burden of mesothelioma deaths in many African countries is likely to be much higher than is seen in referenced data ^12^. Diagnosis of the disease is often complicated and delayed because MPM often presents with vague symptoms such as pleuritic chest pain, dyspnea, or weight loss or a combination of these. On chest X-rays, relatively large amounts of pleural fluid may be found as well ^4, 13^. Despite advancements in treatment modalities, the prognosis of malignant mesothelioma is poor; median overall survival rates of epithelioid MPM are between 12 and 27 months after diagnosis. Sarcomatoid and biphasic mesotheliomas have even poorer prognoses than the epithelioid subtype (7–18 months and 8–12 months, respectively) ^14^. 

Standard work-up for MPM consists of a chest X-ray, computed tomography (CT) scan of the chest and upper abdomen, complete laboratory blood tests, and a thoracentesis with cytological examination of the pleural effusion. CT scans are often combined with positron emission tomography scans to localize possible metastases and to evaluate treatment responses ^3, 8^. Correct staging of MPM is an important step in determining which treatment modalities would be most effective and appropriate. The tumor, node, and metastasis (TNM) classification as proposed by the International Mesothelioma Interest Group in 1994 was widely used for the staging of MPM. In 2016, the International Association for the Study of Lung Cancer (IASLC) published a revised version of this classification on the basis of a large-scale analysis of an international database of patients. A number of important revisions have been made with regard to the different staging components.

In the eighth TNM classification, tumor stages T1a and T1b have been combined into one category, T1. This revision implies that no longer will a distinction be made between tumors confined only to the parietal (T1a) or with extension to the visceral pleura (T1b). Statistical analyses of the IASLC database of patients with T1a and T1b showed no significant difference in overall survival. As for the N component, a number of changes were proposed as well. Both ipsilateral intrapleural and extrapleural nodes have been grouped as N1 disease where previously these were considered separate categories (N1 and N2). Furthermore, the components of N3 disease have been shifted and now are defined as N2 disease. Both pN1 and pN2 are still considered stage III disease. For the M descriptors, no changes have been proposed for its components; however, only M1 is now considered stage IV disease ^15– 18^.

There are a number of possible treatment modalities for MPM, and the choice of treatment strategy is based on patient-related and disease-related factors. The most common treatment options are surgical resection, chemotherapy, radiation therapy (RT), and immunotherapy. Often a multimodal approach is preferred to increase treatment effectiveness and to obtain an optimal survival rate. Multimodality treatment can be applied in both a curative and a palliative setting ^8, 19^.

## Surgical resection

Surgery for MPM is indicated mainly in multimodal approaches and in clinical trial settings according to the latest guidelines. In general, there are two main approaches for surgery with radical and curative intent: (extended) pleurectomy/decortication (P/D) and extrapleural pneumonectomy (EPP) ^8, 20^. Initially, these two surgical approaches did not have clear definitions and the extent of these procedures varied greatly between surgeons. Recently, the IASLC proposed a number of definitions for EPP and P/D:

•   EPP: en bloc removal of the lung, the parietal and visceral pleura, diaphragm, and pericardium

•   Extended P/D: the same procedure as EPP but the lung is left *in situ*

•   P/D: removal of all gross tumor without resection of the diaphragm or the pericardium

•   Partial pleurectomy: a partial resection of parietal or visceral pleura or both without removal of all gross tumor ^15^

Whether EPP or (extended) P/D is the superior approach for malignant mesothelioma is a hotly debated topic. For a long time, EPP was the most widely used approach because it was considered the only way to obtain a macroscopic complete resection. It was hypothesized that EPP was less likely to leave residual tumor cells when compared with P/D; however, more recent studies have shown that in many cases neither EPP nor P/D results in complete R0 resections ^21^. In recent years, there has been a shift in preference of surgeons toward an extended P/D instead of EPP. This trend has been supported by a number of studies that have shown significantly lower complication rates, lower peri-operative morbidity and mortality with P/D, and similar (if not superior) overall survival rates ^22^. Moreover, postoperative quality of life (QoL) seems to be worse in patients after EPP when compared with P/D because of higher rates of complications such as pleural empyema and bronchopleural fistula ^23, 24^.

## Chemotherapy

Chemotherapy plays an important role in the management of MPM. The standard systemic therapy consists of a combination of cisplatin and pemetrexed or cisplatin and raltitrexed ^19^. In some cases, such as in elderly patients, carboplatin may be used as a valid alternative to cisplatin ^25^. Cisplatin interferes with DNA replication, and pemetrexed is a folate antimetabolite that works by interfering with nucleic acid synthesis ^19, 26^. Peri-operative chemotherapy is used for MPM with the goal of increasing local and systemic control of the disease. Furthermore, cisplatin and pemetrexed are used as a pre-operative (induction) treatment to decrease tumor volume and to increase the chances of a more complete resection. The combination of cisplatin and pemetrexed has been used in the majority of clinical studies and has also been combined with surgery and RT in evaluating trimodality therapies ^3, 8, 19^. A number of single-arm phase II studies have investigated the effects of trimodality therapy and have shown promising results ^27, 28^. However, despite these results, data from the National Cancer Database (NCDB) show that the frequency of trimodality therapy has not increased significantly in the US, even though the use of chemotherapy with or without surgery has increased ^29^. As second-line treatment, chemotherapeutic agents such as gemcitabine, vinorelbine, and pemetrexed are used as well, even though evidence is lacking. In the recent LUME-Meso study, the combination of nintedanib and cisplatin/pemetrexed was evaluated in a phase II randomized, placebo-controlled trial. The results showed an improvement in progression-free survival for the treatment arm, and recruitment for a phase III trial has already started ^30^. Another recent randomized controlled study focusing on second-line therapy is the MAPS-2 trial, in which patients with progression after first-line cisplatin/pemetrexed were randomly assigned to one of two groups. One group received nivolumab monotherapy and the other group a combination of nivolumab and ipilimumab. The primary endpoint was disease control rate at 12 weeks, and both treatment arms of this trial reached this endpoint ^31^.

## Radiation therapy

RT is not (yet) considered a standard treatment in the setting of MPM, and literature on this topic is relatively scarce. The aim of RT is to maximize tumor control rates with minimal damage to surrounding normal tissue. It can also be used as an adjuvant therapy after surgery or as part of a trimodality approach. For example, after non-lung-sparing surgery (EPP), hemithoracic adjuvant RT may be offered to patients ^8, 32^. Moreover, in a palliative setting, RT can be applied to reduce chest wall pain ^33^. Technological developments in this field such as intensity-modulated radiotherapy may increase the role of RT in the treatment of MPM. The IMPRINT and SMART trials are examples of a possible and safe therapeutic benefit of RT within a multimodal approach ^34^. However, owing to a lack of large clinical studies with definitive results, the role of RT largely remains within the settings of clinical trials and palliation ^8, 19, 35^.

## Immunotherapy

Immunotherapy plays an increasingly important role in the management of malignancies. It acts by inhibiting immune checkpoints such as cytotoxic T-lymphocyte antigen 4 (CTLA-4) or programmed cell death protein-1 (PD-1) and its ligand (PD-L1) ^36, 37^. Some early clinical phase studies have evaluated the effect of immune checkpoint inhibitors in the management of MPM. The MESOT-TREM-2008 and MESOT-TREM-2012 are two single-center and single-arm trials that have evaluated the effect of tremelimumab, an anti-CTLA-4 monoclonal antibody. In these two trials, clinical activity and acceptable safety profiles were found in MPM patients with progression after chemotherapy ^38, 39^. However, in the multicenter, randomized, placebo-controlled DETERMINE trial, no significant difference in overall survival and progression-free survival was found between the treatment and placebo groups. The researchers of this trial noted that although monotherapy did not result in a survival benefit, combined CTLA-4 and PD-L1 blockade could result in additional therapeutic benefit. Regarding PD-L1 blockade, a number of important trials have been published or are ongoing. The KEYNOTE-028 trial has shown that the anti-PD-L1 monoclonal antibody pembrolizumab has clinical benefit in a proportion of patients with PD-L1-positive MPM ^40^. This has resulted in several phase II trials such as the KEYNOTE-158 basket trial (ClinicalTrials.gov Identifier: NCT02628067), which aims at assessing biomarkers of pembrolizumab response, and the KEYNOTE-139 study (ClinicalTrials.gov Identifier: NCT02399371), which assesses the activity of fixed-dose pembrolizumab as a second-line therapy for MPM. The NIBIT-MESO-1 trial is one of the studies that are designed to investigate the possible therapeutic benefit of combining immunotherapies. This phase II study combines tremelimumab with durvalumab, an anti-PD-L1 monoclonal antibody. In this single-center, non-randomized study, the results seem promising with an acceptable safety profile ^41^. Other examples of ongoing trials that are investigating the combination value of PD-1/PD-L1 and CTLA-4 blockade are the previously mentioned MAPS-2 trial and Checkmate 743 ^31, 42^.

## Multimodal treatments

A number of non-randomized clinical trials have investigated the feasibility and outcomes of trimodality treatments for MPM. Rea *et al*. have evaluated the effects of induction chemotherapy with three or four cycles of carboplatin/gemcitabine, EPP, and postoperative RT in a prospective study with 21 patients ^43^. Their results showed a median overall survival rate of 25.5 months and demonstrated that a combined multimodal approach is feasible ^43^. Another trial investigating trimodality therapy was published by Yamanaka *et al*. ^44^. In this phase II study, 42 patients were enrolled and treated with induction chemotherapy using cisplatin/pemetrexed, EPP, and postoperative hemithoracic RT. In total, 17 patients received trimodality therapy and the overall median survival time for this group was 39.4 months. The overall median survival time for all 42 patients who were enrolled in the study was 19.9 months. However, relapse patterns were similar for patients with or without postoperative RT in this trial. The authors have emphasized that although trimodality therapy seemed feasible, the risk-to-benefit ratio was unsatisfactory ^44^. A similar study was set up by the European Organisation for Research and Treatment of Cancer (EORTC) and 59 patients were enrolled. In total, 37 patients received trimodality therapy consisting of induction chemotherapy with cisplatin/pemetrexed, EPP, and postoperative RT. In this study, trimodality treatment was deemed feasible in selected patients with early stage mesothelioma when used in institutions with high levels of expertise and in prospective clinical trials ^28^.

Until now, two randomized controlled trials have evaluated the effects of multimodality treatments. In the first trial, Treasure *et al*. compared the effects of EPP plus postoperative hemithoracic RT versus standard (non-radical) therapy alone following platinum-based chemotherapy. In this MARS 1 study, 50 patients were deemed eligible for randomization: 24 patients were assigned to the EPP arm and 26 to the standard therapy arm. In the end, 16 out of 24 patients completed their treatment with EPP. The median overall survival in the EPP arm was 14.4 months from randomization, and in the non-EPP arm the overall survival was 19.5 months. Furthermore, the EPP arm was also associated with higher morbidity and more serious adverse events than the non-EPP arm. The authors suggested after these findings that a radical approach with EPP offered no benefit when compared with a non-radical approach ^45^.

The second randomized clinical trial evaluated the effects of hemithoracic radiotherapy after neoadjuvant chemotherapy and EPP. In total, 54 patients were deemed eligible for randomization: 27 patients were assigned to the RT arm and 27 to the non-RT arm. The overall median survival in both groups was about 20 months. Furthermore, QoL in both patient groups was evaluated, and patients in the non-RT arm reported improvements in physical and psychological symptom distress and activity impairment. After their treatment, patients enrolled in the RT arm reported stable scores in these domains except for activity impairment, which worsened in the first 4 weeks following randomization; however, this stabilized to baseline scores afterwards. In general, their findings did not support the routine use of hemithoracic RT after neoadjuvant chemotherapy and EPP ^46^.

Currently, a number of ongoing trials are investigating the effects of multimodal therapies. MARS 2 (ClinicalTrials.gov Identifier: NCT02040272) is a randomized controlled trial that compares the outcomes of platinum-based chemotherapy plus P/D versus chemotherapy alone. Another clinical study currently being conducted is the EORTC 1205 trial (ClinicalTrials.gov Identifier: NCT02436733), a phase I randomized trial that compares an immediate surgery arm consisting of P/D followed by three cycles of cisplatin/pemetrexed with delayed surgery starting with the same chemotherapeutic regimen followed by P/D. These trials differ from the previously discussed studies that have incorporated surgery in the multimodal approach in that, in the former, P/D instead of EPP is used as surgical treatment ^19^.

In the near future, immunotherapy may play a larger part in the multimodal treatment approach of MPM as well. Currently, the combination of immunotherapy, surgical resection, and chemotherapy is also being investigated in an ongoing trial (ClinicalTrials.gov Identifier: NCT02707666). In this single-center, single-arm trial, pembrolizumab is administered in three cycles followed by P/D and postoperative chemotherapy with cisplatin/pemetrexed. Another ongoing trial (ClinicalTrials.gov Identifier: NCT02592551) is investigating the effects of durvalumab and durvalumab plus tremelimumab before resection surgery (EPP or P/D) in a randomized controlled trial. A number of other promising treatment modalities are currently being investigated within a multimodal approach, such as photodynamic therapy (ClinicalTrials.gov Identifier: NCT02662504), arginine deprivation (ClinicalTrials.gov Identifier: NCT02709512), and immunogene therapy (ClinicalTrials.gov Identifier: NCT01119664).

## Discussion

Multimodality treatment for MPM is a topic that has been attracting a lot of attention from researchers in the last couple of years, as therapeutic modalities such as surgery, chemotherapy, or radiotherapy have not proven to be effective as single-modality treatments. Many studies have investigated the effects of multimodal approaches; however, in many cases, the quality of published evidence is poor and underpowered ^28, 47^. In recent years, larger, multicenter, randomized trials have been started with the aim of reaching a consensus on effective treatment approaches. These studies induced a number of trends (for example, the shift from more radical approaches such as EPP to more conservative treatments such as lung-sparing surgery [P/D] ^21^). Even randomized controlled trials such as those by Treasure *et al*. ^45, 46^ and Stahel *et al*. ^45, 46^ do not provide definite answers; in fact, paradoxically, they add to the surgical controversy resulting in ongoing debates at international conferences.

One of the main issues in studies regarding surgery for MPM is the selection bias that is often present. For the vast majority of patients with MPM, the choice for surgery is based on a large number of criteria such as histology, performance status, age, tumor invasiveness, and tumor bulk. For clinical researchers, this selection bias makes it much more difficult to provide unbiased results from clinical studies ^48^. Although there is evidence that cancer-directed surgery for MPM provides a survival benefit, researchers agree that patients should be carefully selected and be considered for clinical trial enrollment ^49^. A number of ongoing randomized controlled trials are investigating the role of multimodal treatments with less-invasive surgery (P/D). The results from these trials may prove to be valuable in establishing a definitive role for chemotherapy, RT, and lung-sparing surgery in the treatment of MPM.

Furthermore, trials including immunotherapy such as pembrolizumab, tremelimumab, and durvalumab represent a brand-new area that very likely will be a topic of interest in the next decade. As of now, a number of ongoing trials have combined immunotherapy with chemotherapy or surgery or both. The DREAM trial is an example of a phase II study evaluating the effects of immunotherapy, more specifically durvalumab, combined with cisplatin/pemetrexed. New therapeutic approaches for MPM are on the rise as well. Treatment modalities such as microRNA-loaded minicells targeting epidermal growth factor receptor (EGFR) for tumor suppression, Wilms’ tumor 1 vaccines, and tyrosine kinase inhibitors could prove to be valuable additions to the standard therapeutic approaches for MPM ^34, 50^.

Besides research into treatment modalities for MPM, developments in tumor measurement and response assessment are critically important. Recently, the revised mRECIST (modified response evaluation criteria in solid tumors) were published ^51^. These criteria help in establishing international guidelines for the measurement of tumors and response assessments for MPM. With these guidelines, inaccuracies and inconsistencies across clinical trials will be minimized. This allows results from clinical studies to be more robust and uniform, thus allowing more precise comparisons between different studies. Hopefully, these new advances in imaging, staging, and multimodality therapy will improve the grim prognosis of MPM without compromising the QoL of these patients, who still have a limited life expectancy.
